# Safety and microbiological activity of phage therapy in persons with cystic fibrosis colonized with *Pseudomonas aeruginosa:* study protocol for a phase 1b/2, multicenter, randomized, double-blind, placebo-controlled trial

**DOI:** 10.1186/s13063-022-07047-5

**Published:** 2022-12-28

**Authors:** Pranita D. Tamma, Maria Souli, Michael Billard, Joseph Campbell, Douglas Conrad, Damon W. Ellison, Beth Evans, Scott R. Evans, Kerryl E. Greenwood-Quaintance, Andrey A. Filippov, Holly S. Geres, Toshimitsu Hamasaki, Lauren Komarow, Mikeljon P. Nikolich, Thomas P. Lodise, Seema U. Nayak, Carmelle Norice-Tra, Robin Patel, David Pride, Janie Russell, Daria Van Tyne, Henry F. Chambers, Vance G. FowlerJr, Robert T. Schooley, for the Antibacterial Resistance Leadership Group

**Affiliations:** 1 Department of Pediatrics, Johns Hopkins University School of Medicine, 200 North Wolfe Street, Room 3149, Baltimore, MD 21287 USA; 2 Duke Clinical Research Institute, Duke University Medical Center, Durham, NC USA; 3 Adaptive Phage Therapeutics, Inc., Gaithersburg, MD USA; 4 National Institutes of Health, National Institute of Allergy and Infectious Diseases, Division of Microbiology and Infectious Diseases, Bethesda, MD USA; 5 Department of Medicine, University of California San Diego, San Diego, CA USA; 6 Wound Infections Department, Bacterial Diseases Branch, Walter Reed Army Institute of Research, Silver Spring, MD USA; 7 The Biostatistics Center, The George Washington University, Rockville, MD USA; 8 Department of Laboratory Medicine and Pathology, Mayo Clinic, Rochester, USA; 9 Department of Pharmacy Practice, Albany College of Pharmacy and Health Sciences, Albany, NY USA; 10 Infectious Diseases and Occupational Medicine, Mayo Clinic, Rochester, MN USA; 11 Departments of Medicine and Pathology, University of California San Diego, San Diego, CA USA; 12 Department of Medicine, University of Pittsburgh, Pittsburgh, PA USA; 13 Department of Medicine, University of California San Francisco, San Francisco, CA USA; 14 Department of Medicine, Duke University Medical Center, Durham, NC USA

**Keywords:** Phage, Multidrug-resistant, *Pseudomonas aeruginosa*, Cystic fibrosis

## Abstract

**Background:**

Bacteriophages (phages) are a promising anti-infective option for human disease. Major gaps remain in understanding their potential utility.

**Methods:**

This is a randomized, placebo-controlled, double-blind study of a single dose of intravenous phage in approximately 72 clinically stable adult cystic fibrosis volunteers recruited from up to 20 US sites with *Pseudomonas aeruginosa* airway colonization. The single dose of phage consists of a mixture of four anti-pseudomonal phages. Six sentinel participants will be sequentially enrolled with dose escalation of the phage mixture by one log_10_ beginning with 4 × 10^7^ plaque-forming units in an unblinded stage 1. If no serious adverse events related to the study product are identified, the trial will proceed to a double-blinded stage 2. In stage 2a, 32 participants will be randomly assigned to one of three phage dosages or placebo in a 1:1:1:1 allocation. An interim analysis will be performed to determine the phage dosage with the most favorable safety and microbiological activity profile to inform phage dosing in stage 2b. During stage 2b, up to 32 additional volunteers will be randomized 1:1 to the phage or placebo arm. Primary outcomes include (1) the number of grade 2 or higher treatment-emergent adverse events, (2) change in log_10_ *P. aeruginosa* total colony counts in sputum, and (3) the probability of a randomly selected subject having a more favorable outcome ranking if assigned to receive phage therapy versus placebo. Exploratory outcomes include (1) sputum and serum phage pharmacokinetics, (2) the impact of phage on lung function, (3) the proportion of *P. aeruginosa* isolates susceptible to the phage mixture before and after study product administration, and (4) changes in quality of life.

**Discussion:**

This trial will investigate the activity of phages in reducing *P. aeruginosa* colony counts and provide insights into the safety profile of phage therapy.

**Trial registration:**

ClinicalTrials.gov NCT05453578. Registered on 12 July 2022.

## Administrative information

Note: the numbers in curly brackets in this protocol refer to SPIRIT checklist item numbers. The order of the items has been modified to group similar items (see https://nam02.safelinks.protection.outlook.com/?url=http%3A%2F%2Fwww.equator-network.org%2Freporting-guidelines%2Fspirit-2013-statement-defining-standard-protocol-items-for-clinical-trials%2F&data=05%7C01%7Cptamma1%40jhmi.edu%7C8a9070b40c9b4e30013508dae2956940%7C9fa4f438b1e6473b803f86f8aedf0dec%7C0%7C0%7C638071428605335766%7CUnknown%7CTWFpbGZsb3d8eyJWIjoiMC4wLjAwMDAiLCJQIjoiV2luMzIiLCJBTiI6Ik1haWwiLCJXVCI6Mn0%3D%7C3000%7C%7C%7C&sdata=YeTLV3DMVzhTG2PVNOyQ4DqVrMRP0qJ%2F54u5goMDS5k%3D&reserved=0).

**Table:** 

^**1**^ **Title {1}**	A Phase 1b/2, Multi-Centered, Randomized, Double-Blind, Placebo-Controlled Trial of the Safety and Microbiological Activity of a Single Dose of Phage Therapy in Cystic Fibrosis Volunteers Colonized with *Pseudomonas aeruginosa*
**Trial registration {2a and 2b}.**	ClinicalTrials.gov register identifier: NCT05453578
**Protocol version {3}**	Protocol number 20-0001, version 4.0, 7 October 2022
**Funding {4}**	National Institutes of Health (NIH), Grant: 5UM1Al104681-09
**Author details {5a}**	As above
**Name and contact information for the trial sponsor {5b}**	National Institute of Allergy and Infectious Diseases (NIAID) Bethesda, Maryland 20892
**Role of sponsor {5c}**	The study sponsor is the NIAID. The research team is responsible for study design, data management, analysis, manuscript preparation, and disseminating trial findings.

## Introduction

### Background and rationale {6a}

Multidrug-resistant gram-negative (MDRGN) bacterial infections remain an international public health concern [1]. The cystic fibrosis (CF) population is prone to respiratory infections (i.e., pulmonary exacerbations) caused by MDRGN pathogens [2]. These manifest as lower respiratory tract infections that are increasingly challenging to treat as resident bacteria accumulate increasing levels of drug resistance while remaining protected in biofilms that further compromise antibiotic effectiveness [2]. *Pseudomonas aeruginosa* is the most common pathogen responsible for CF exacerbations [2]. Because of the limited number of antibiotics available to treat pulmonary exacerbations caused by organisms such as *P. aeruginosa* in persons with CF, novel treatment approaches are needed [3].

Bacteriophages (herein, referred to as phages) are viruses that target and kill bacteria [4]. Since the 1940s, with the advent of modern antibiotics, their use as antimicrobial therapeutics in the Western world was largely abandoned. In recent years, phage administration as an adjunct therapy to systemic antibiotics for the treatment of complex infections under compassionate use conditions has increased, as is evident by a growing number of case reports and case series in the literature [5–7]. These clinical experiences have noted very few safety concerns [7].

Unique features that make phages attractive for clinical use include their bactericidal activity [4, 8], high specificity for target pathogens including MDRGN organisms [8], the ability to penetrate eukaryotic cells and target intracellular bacteria [8], amplification in vivo in the presence of target bacteria [9], biofilm penetration and antibiofilm activity [10], avoidance of host tissue damage [8, 9], preservation of the human microbiome (compared with antibiotics) [11], and frequent synergy with antibiotics [12]. However, rigorous scientific investigations are required to address critical gaps in knowledge before phage therapy can be routinely used in clinical practice. The overarching goal of the proposed clinical trial is to enhance the understanding of the safety and microbiologic activity of phage therapy as anti-infectives.

### Objectives {7}

#### Primary objectives

- Describe the safety of a single dose of intravenous (IV) phage therapy in CF volunteers with *P. aeruginosa* in expectorated sputum
- Describe the microbiological activity of a single dose of IV phage therapy in CF volunteers with *P. aeruginosa* in expectorated sputum
- Describe the benefit-to-risk profile of a single dose of IV phage therapy in CF volunteers with *P. aeruginosa* in expectorated sputum

#### Exploratory objectives

- Characterize the serum and sputum pharmacokinetic profiles of a single dose of IV phage therapy in CF volunteers with *P. aeruginosa* in expectorated sputum
- Describe changes in lung function in CF volunteers after the administration of a single dose of IV phage therapy
- Characterize phage susceptibility among geographically diverse *P. aeruginosa* isolates from CF volunteers
- Describe CF volunteers’ quality of life (QoL) before and after receiving phages

### Trial design {8}

This is a phase 1b/2, multicenter, randomized, placebo-controlled, double-blind study of a single dose of IV phage in approximately 72 clinically stable adult CF volunteers colonized with *P. aeruginosa* in expectorated sputum. The primary study outcomes include the safety and microbiological activity of IV phage therapy. The single dose of IV phage will consist of a mixture of four anti-pseudomonal phages.

**Fig. 1:**
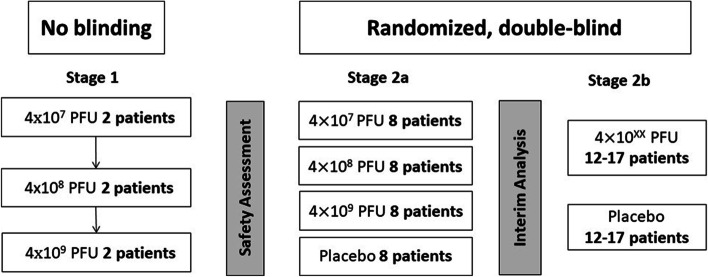
Study design

Stage 1 will involve six volunteers assigned to one of three IV phage dosing arms (Fig. 1). The arms will be sequentially enrolled with dose escalation by one log_10_ starting at 4 × 10^7^ plaque-forming units (PFU). In each dosing arm, two volunteers will be enrolled. Each sentinel subject will receive a single dose of IV phage therapy. If no serious adverse events (SAEs) related to the study product are identified during the 96 h after phage administration in stage 1 volunteers, the study will proceed to stage 2.

In stage 2a, 32 volunteers will be randomly assigned to one of three IV phage doses or placebo in a 1:1:1:1 allocation. Volunteers and the protocol team will be blinded to phage versus placebo preparations. An interim analysis will be performed to determine the phage dosing with the most favorable safety and microbiological activity profile after 8 volunteers per arm have been randomized and completed their last follow-up visit at day 30. The interim analysis identifies the phage dose and the sample size that will be used for stage 2b. During stage 2b, up to 34 additional volunteers will be randomized into a phage or placebo arm. The final trial sample size is expected to be up to 72 volunteers.

## Methods: participants, interventions, and outcomes

### Study setting {9}

Clinically stable volunteers will be recruited from up to 20 CF centers in the USA. Study visits will occur in the ambulatory setting. A list of study sites can be obtained from Clinicaltrials.gov NCT05453578.

### Eligibility criteria {10}

Volunteers must meet all inclusion criteria to be eligible to enroll in the study:

- Adult (≥ 18 years) at the time of screening
- CF diagnosis based on a compatible clinical syndrome confirmed by either abnormal sweat chloride testing or cystic fibrosis transmembrane conductance regulator gene variations
- Ability to produce approximately 2 mL of sputum over a 30-min period
- *P. aeruginosa* isolated from a sputum culture, throat culture, or other respiratory specimens in the past 12 months
- Confirmed *P. aeruginosa* isolation from a sputum sample at the screening visit
- Capable of providing informed consent
- Capable and willing to complete all study visits and perform all procedures required by the protocol

Volunteers who meet any of the following exclusion criteria will not be enrolled in the study:

- Body weight <30 kg
- Forced expiratory volume in 1 s (FEV1) <20% of predicted at screening
- Elevated liver function tests obtained at screening
- Acute clinical illness requiring a new oral, parenteral, or inhaled antibiotic(s) ≤30 days prior to the baseline visit
- Pregnant, planning to become pregnant during the study period, or breastfeeding
- Active treatment of any mycobacteria or fungal organisms ≤30 days prior to the baseline visit
- Anticipated need to change chronic antibiotic regimens during the study period
- Known allergy to any component of the study product
- Any significant finding that, in the opinion of the investigator, would make it unsafe for the volunteer to participate in the study
- Enrollment in a different clinical trial ≤30 days of the baseline visit, or while enrolled in the current clinical trial
- Previous enrollment in the current trial

### Who will obtain informed consent? {26a}

Upon identification of a potentially eligible participant, study procedures, risks, and potential benefits will be presented by the local study team during a screening visit. Participants will receive a copy of the study consent and will have the opportunity to ask questions. Before any study procedures are performed, informed consent will be obtained and documented.

### Additional consent provisions for collection and use of participant data and biological specimens {26b}

The information and specimens collected for this study may be used for future research. The research may include, but is not limited to, investigating the role of serum neutralizing antibodies, whole genome sequencing of *P. aeruginosa* isolates to identify changes that occur after exposure to phages, and the impact of phages on the respiratory microbiome. No human genetic testing (i.e., sequencing of human DNA) will be performed.

## Interventions

### Explanation for the choice of comparators {6b}

This trial will compare the safety and microbiological activity of phages versus placebo in clinically stable CF volunteers with chronic *P. aeruginosa* airway colonization. Phage therapy is not currently approved as an anti-infective for use in humans by the US Food and Drug Administration (FDA). As such, most humans who have received phages as anti-infectives have received them in conjunction with, rather than in place of, antibiotic therapy [6]. This complicates the assessment of the independent role of phages on reducing bacterial colony counts in humans. Additionally, notable proportions of patients who have previously received phage therapy had comorbidities, clinical instability, and/or simultaneously received several other medications, rendering our understanding of the adverse event (AE) profile attributable to phage therapy incomplete [7]. An understanding of the independent microbiological activity and safety profile of phages is essential to furthering the science of phage therapy for human infections.

### Intervention description {11a}

The product used in this study is WRAIR-PAM-CF1. WRAIR-PAM-CF1 is a cocktail of four phages in a 1:1:1:1 combination: PaWRA01Phi11, PaWRA01Phi39, PaWRA02Phi83, and PaWRA02Phi87 (formerly WRAIR_EPa11, WRAIR_EPa39, WRAIR_EPa83, WRAIR_EPa87), all of which are lytic against *P. aeruginosa*. These phages are naturally occurring and free of known deleterious genes, including genes essential for lysogeny (i.e., they cannot incorporate into the chromosome of the bacterial host), antibiotic resistance genes, and toxin genes*.* Each phage lot has been manufactured by Adaptive Phage Therapeutics (APT) in accordance with current Good Manufacturing Practices. Endotoxin levels in the phage lots are below acceptable limits set by the FDA (5 endotoxin units/kg/h). The phage mixture is administered as a total of 4 × 10^7^ PFU, 4 × 10^8^ PFU, or 4 × 10^9^ PFU, depending on the target dose. The final phage combination to be administered to a trial participant is diluted to the target dose in normal saline. The minimum and maximum phage doses that are being investigated were informed by previous case reports, case series, and clinical trials, which generally used IV doses ranging from approximately 10^7^ PFU to 10^10^ PFU per dose [6, 7]. All phage and placebo preparation will occur in an investigational pharmacy by an unblinded pharmacist and will be aliquoted for equal volume administrations to maintain the double-blind design.

### Criteria for discontinuing or modifying allocated interventions {11b}

Volunteers may withdraw consent for study participation at any time without penalty. An investigator may also withdraw a volunteer from receiving the study product if it is determined that participation in the study is not in the best interest of the subject. Follow-up safety evaluations will be conducted, if the volunteer agrees.

If any of the following events occur, enrollment and dosing for all volunteers will be suspended until the event is assessed by the Data Safety and Monitoring Board (DSMB): (1) any volunteer develops an SAE related to the study product through the last study visit, (2) two or more volunteers in the study experience a grade 3 or higher AE that is related to study product and is of the same type through the last study visit, (3) any volunteer develops anaphylaxis within 24 h after receiving the study product, or (4) any volunteer reports two or more pulmonary exacerbations, from the time of study product administration through day 8. Moreover, an individual infusion will be stopped if a drug-related hypersensitivity reaction is suspected. Study withdrawal could also occur if the study or study site is terminated by the sponsor for any reason.

### Strategies to improve adherence to interventions {11c}

Recruitment of volunteers will occur by site investigators who are clinicians who care for persons with CF. Although there are no prespecified strategies that have been developed to increase adherence, only volunteers willing and able to participate in all planned follow-up visits will be selected for trial inclusion.

### Relevant concomitant care permitted or prohibited during the trial {11d}

Concomitant medications will be reviewed during each trial visit. If a volunteer is prescribed antibiotic therapy with activity against *P. aeruginosa* after receipt of the study product, the volunteer will continue in the trial and be included in an intent-to-treat analysis. Receipt of antibiotic therapy with activity against *P. aeruginosa* will be documented as a concomitant medication and the underlying condition for which the antibiotic was taken will be reported as an AE. Chronic medications, rescue medications, and over-the-counter medications are allowed.

### Provisions for post-trial care {30}

All volunteers will be followed for 30 days. AEs will be assessed and followed from initial recognition of the AE through the end of the 30-day follow-up period. SAEs will be followed through resolution even if the duration of follow-up goes beyond the planned follow-up period.

### Outcomes {12}

**Fig. 2:**
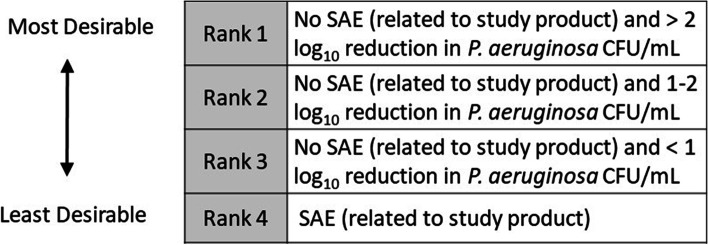
Desirability of outcome ranking. SAE severe adverse event, CFU colony forming units

Three outcomes will be analyzed to investigate the primary objectives: (1) the number of grade 2 or higher treatment-emergent AEs through the day 30 visit, (2) change from baseline to day 30 log_10_ *P. aeruginosa* total colony counts in sputum cultures after administration of study product, and (3) desirability of outcome ranking (DOOR) using the greatest reduction by the day 8 visit. More specifically, volunteers will be placed into one of four DOOR categories (Fig. 2). The DOOR will be analyzed by estimating the probability of a randomly selected subject having a better DOOR if assigned to receive phage therapy compared to placebo.

Several exploratory endpoints will also be investigated: (1) sputum and serum pharmacokinetics of phage therapy, (2) impact on FEV1 from the administration of study product through day 30, (3) the proportion of *P. aeruginosa* isolates susceptible to the four individual phages and the phage mixture before and after exposure to study product, and (4) changes in QoL of participants based on responses documented in the Cystic Fibrosis Questionnaire-Revised and the Cystic Fibrosis Respiratory Symptom Diary before and after exposure to the study product.

### Participant timeline {13}

**Fig. 3:**
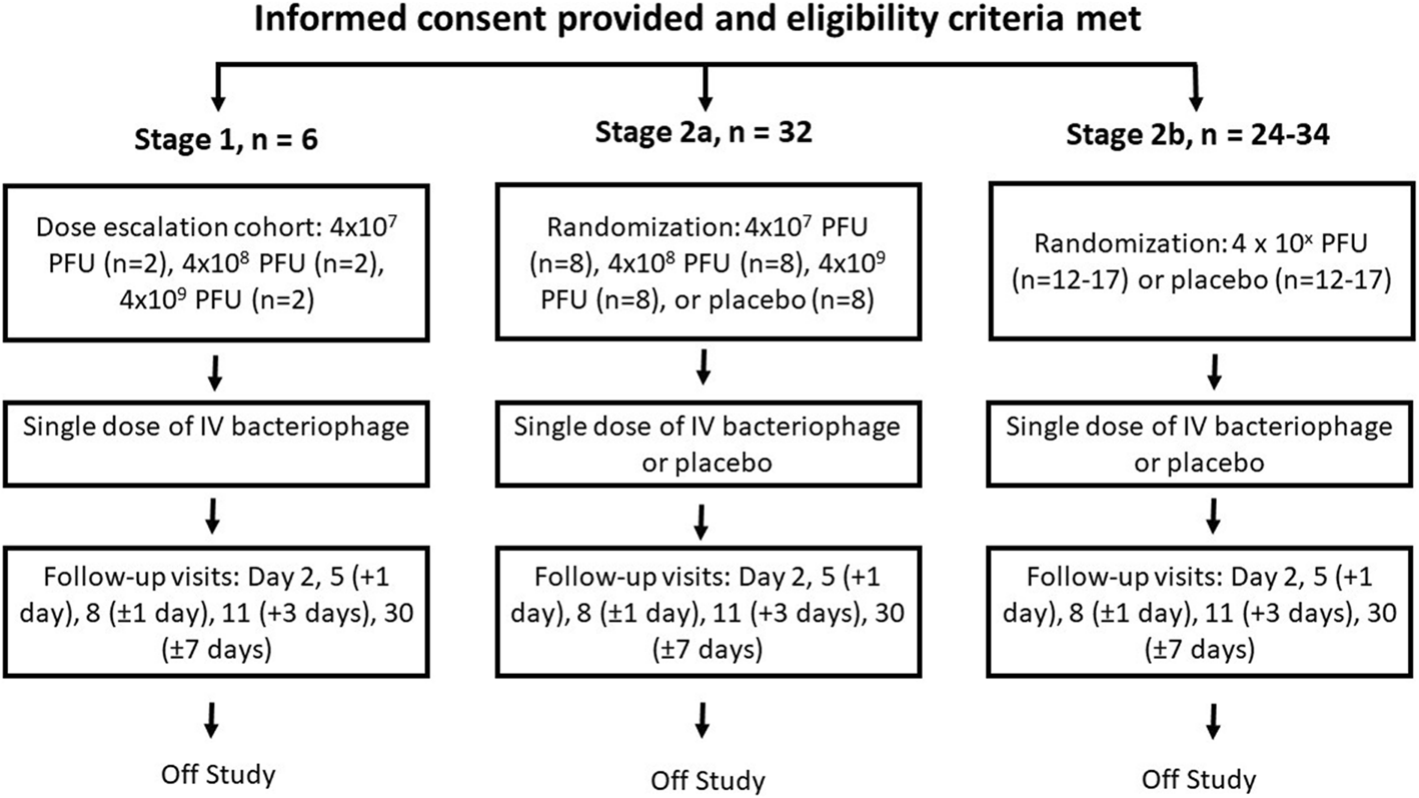
Participant timeline

The participant timeline is illustrated in Fig. 3.

### Sample size {14}

The sample size was calculated to provide desired precision of the estimate of the DOOR probability in order to describe the benefit-to-risk profile of a single dose of IV phage. If the DOOR probability comparing IV phage and placebo is 70%, when the total sample size in each arm is 20 (combining volunteers from stages 2a and 2b), the two-sided normal approximate 95% confidence interval for DOOR probability is calculated at 51% and 89%, respectively, with the lower limit larger than 50%. Superiority will be considered to have been achieved if the 95% confidence interval for the probability does not cross 50%.

Based on the interim analysis after stage 2a, the planned sample size for stage 2b will be re-evaluated as to whether it provides the desired precision of estimates of the DOOR probability for a selected phage dose and placebo. The total estimated sample size for the trial is 72 participants. In the intention-to-treat population, it is estimated that there will be up to 25 volunteers in the phage arm (for the final selected dose) and 25 volunteers in the placebo arm.

### Recruitment {15}

CF volunteers will be recruited from up to 20 outpatient clinics in the USA. There will be no enrollment from international sites. It is anticipated that approximately three patients will be enrolled per month. The anticipated enrollment period is from October 3, 2022, until January 31, 2024.

## Assignment of interventions: allocation

### Sequence generation {16a}

In stage 2a, volunteers will be randomized to either one of three IV phage doses or placebo with a 1:1:1:1 allocation as per a computer-generated randomization stratified by site, using a permuted block design. In stage 2b, volunteers will be randomly assigned to one of two arms: the selected phage dose (after interim analysis) or placebo in a 1:1 allocation. The block size will be concealed until the primary endpoint is analyzed.

### Concealment mechanism {16b}

Volunteers will be randomized using the Advantage eClinical data management system, a centralized, web-based enterprise resource developed and maintained by the Emmes Company. Allocation concealment will be ensured, as the randomization code will not be released until the patient has been recruited into the trial. The codes will be kept confidential and allocation communicated to sites electronically via a separate online enrollment module.

### Implementation {16c}

The randomization process will be managed via an online enrollment module within the Advantage eClinical data management system.

## Assignment of interventions: blinding

### Who will be blinded {17a}

In stage 2, the subject and the investigators will be unaware of treatment group assignments. The three phage doses and placebo will be packaged identically so that treatment blinding is maintained. Specimens provided to the laboratory for analyses will be blinded to participant identification and visit number in addition to treatment assignment. Only the site pharmacist preparing the study product will be unblinded.

### Procedure for unblinding if needed {17b}

Randomization code breaks will occur only when knowledge of the actual treatment is essential for further management of the subject. Site investigators are encouraged to discuss with the study team and DMID if they believe that unblinding is necessary. In the case of a medical emergency, if the site investigator believes that unblinding would benefit the medical care of the volunteer, the unblinding process can occur on-site by contacting the unblinded pharmacist.

## Data collection and management

### Plans for assessment and collection of outcomes {18a}

Data will be entered electronically by site study staff into Advantage eClinical. Instructions for use of the system and completion of the electronic case report forms (eCRFs) for each study will be included in the Advantage eClinical User’s Guide and the eCRF Instructions. Quality assurance reports will be generated to ensure study data are clean, accurate, and complete. Quality assurance reports will include, but are not limited to, the following: missing forms, missing and out-of-range values, automated data queries, and targeted manual reviews of study data. The schedule of events is described in Table 1.

**Table 1:** Schedule of events

Visit	Screen	Baseline	Follow-up visits					Early termination visit/unscheduled study visit
**Visit number**	**1**	**2**	**3**	**4**	**5**	**6**	**7**	**--**
**Day of visit**	**Up to day −7**	**1**	**2**	**5±2**	**8+1**	**11+7**	**30±7**	**Variable**
Informed consent	X							
Review eligibility criteria	X	X						
Serum HCG pregnancy test	X							
Urine pregnancy test		X						
Demographics	X							
Height and weight	X							
Medical history	X							
Medication history	X							
Review of systems	X	X	X	X	X	X	X	X
Treatment assignment		X						
Administration of study product		X						
Concomitant medications		X	X	X	X	X	X	X
Physical examination	X							
Symptom-directed physical examination		X	X	X	X	X	X	X
Vital signs	X	X	X	X	X	X	X	X
Spirometry	X	X	X	X	X	X	X	X
Sputum collection for microbiology and phage pharmocokinetics	X	X	X	X	X	X	X	X
Clinical chemistry		X	X	X	X	X	X	X
Liver function tests	X	X	X	X	X	X	X	X
Hematology		X	X	X	X	X	X	X
Serum for phage pharmacokinetics		X	X					
Cystic Fibrosis Questionnaire-Revised (stage 2 only)		X					X	
Cystic Fibrosis Respiratory Symptom Diary (stage 2 only)		X	X	X	X	X	X	X
Safety assessment		X	X	X	X	X	X	X
Events of special interest		X	X	X	X			X

### Plans to promote participant retention and complete follow-up {18b}

Volunteers may voluntarily withdraw their consent for study participation at any time without penalty. An investigator may also withdraw a subject from receiving the study product for any reason. Follow-up safety evaluations will be conducted if the subject agrees. Volunteers who withdraw, are withdrawn, or are lost to follow-up after administration of the study product will not be replaced.

### Data management {19}

DMID’s monitoring staff will either conduct site visits or remote source verification to assure appropriate quality and completeness of data. Discrepancies in data entry in eCRFs will trigger data re-entry requirements and/or site retraining for the relevant data fields.

### Confidentiality {27}

Personal health information will be collected and stored securely within the electronic study database maintained by the Emmes Company for a minimum of 2 years after study completion.

### Plans for collection, laboratory evaluation, and storage of biological specimens for genetic or molecular analysis in this trial/future use {33}

Processing of serum chemistries, liver function tests, and hematology to monitor for AEs will occur at local laboratories, in accordance with local standard operating procedures. Sputum specimens will be shipped to a central laboratory for quantification of *P. aeruginosa* by culture and determination of phage concentrations. Phage susceptibility testing (PST) will be performed on cultured *P. aeruginosa* strains. More specifically, sputum will be collected before and after study product administration at the baseline visit, as well as all follow-up visits and processed within 48 h of collection. The total CFU/mL of all morphotypes of *P. aeruginosa* identified in sputum cultures will be evaluated at each visit. As persons with CF are often colonized with more than one morphotype of *P. aeruginosa*, colony size, color, mucoid phenotype, antimicrobial susceptibility testing results, and other morphological features will be documented for each *P. aeruginosa* colony type to determine whether isolates might represent the same *P. aeruginosa* strain over time from the same patient.

From the same sputum specimens and after *P. aeruginosa* quantitative cultures are performed, phage quantitative polymerase chain reaction (qPCR) will be performed to understand the pharmacokinetics of phage in sputum. Phages are expected to amplify upon infecting a susceptible bacterial host (in this case *P. aeruginosa*); therefore, it is hypothesized that the quantity of phage identified in the sputum should increase in the days after a dose of phage is administered.

As the study product is being administered IV, serum pharmacokinetics will also be evaluated. As trial participants would not be expected to have *P. aeruginosa* in their bloodstream, the identification of phage in serum is expected to be short-lived; serum will be analyzed prior to infusion of the study product, and at 30 min, 1 h, 1.5 h, 2 h, 2.5 h, 3 h, and 3.5 h post-administration. Serum PK studies will also be performed at a single timepoint during the day 2 visit (i.e., the day after administration of the study product).

Each morphotype of *P. aeruginosa* obtained from sputum specimens will undergo PST using both index *P. aeruginosa* isolates (i.e., prior to any exposure to the four-phage cocktail) and sequential *P. aeruginosa* isolates identified in sputum samples collected at six timepoints after study product administration, for each subject. PST results will inform the proportion of trial participants with *P. aeruginosa* isolates susceptible to both individual phages and the phage mixture.

As no reference standard testing method for assessing bacterial susceptibility to phages exists, PST will occur using two approaches: (1) plaque assay and (2) liquid assay. Susceptibility to both tests will qualify a *P. aeruginosa* isolate “susceptible” to the phages. Replicate testing will occur for any *P. aeruginosa* isolates with discrepant PST results. Briefly, the plaque assay utilizes a modification of the double agar overlay plaque assay [13]. Phages are grown within a lawn of bacteria and visual evaluation for plaques occurs. The liquid assay is an enhanced version of the Biolog OmniLog® method [14]. The liquid assay monitors phage activity by comparing bacterial metabolic activity in the presence or absence of phage using a tetrazolium dye that monitors bacterial metabolic products in solution. Strict quality control measures will be in place for all sputum and serum laboratory analyses.

## Statistical methods

### Statistical methods for primary and secondary outcomes {20a}

Analyses will be performed on the basis of the intention-to-treat principle. Grade 2 or higher treatment-emergent AEs will be summarized descriptively. The number of events and number and percentage of volunteers with events in each arm will be tabulated and presented by system organ class and likelihood of being related/unrelated. The difference in the proportion of the number and percentage of volunteers with events between phage and placebo arms will be calculated with the corresponding 95% confidence interval.

Changes in log_10_ *P. aeruginosa* CFU/mL (total and each morphology) in quantitative sputum cultures from administration of the study product through day 30 will be summarized descriptively, by treatment arm. The area under the curve (AUC) calculated using the trapezoidal rule will be used to summarize log_10_ *P. aeruginosa* CFU/mL over time. Differences in mean changes in log_10_ *P. aeruginosa* CFU/mL and AUC between the phage and placebo arms will be calculated with the corresponding 95% confidence interval.

The DOOR will be summarized using the DOOR probability [15]. The DOOR probability is estimated by Wilcoxon-Mann-Whitney statistics corrected for ties, divided by the product of the number of volunteers in each group, along with a 95% confidence interval. Given the composite nature of DOOR, individual components will be analyzed and examined separately. Partial credit scoring-based analyses will be conducted.

### Interim analyses {21b}

There will be one planned interim analysis of the primary endpoints after volunteers in stage 2a complete their day 30 follow-up visits. The interim analysis will be performed to select the phage dose with the most favorable benefit-to-risk profile compared to placebo for stage 2b. The interim analysis will consist of a quantitative evaluation of potential effect sizes and associated precision using a predicted interval plots approach that will be generated for a range of assumptions [16]. The results of the interim analysis will not be shared with the site investigators prior to the completion of the trial. The DSMB will advise DMID on whether to continue, modify, or terminate the trial based on risk assessment during the interim analysis.

### Methods for additional analyses (e.g., subgroup analyses) {20b}

As previously stated, there will be four exploratory outcomes. Phage pharmacokinetics will be analyzed using compartmental population pharmacokinetic models to obtain population mean estimates of clearances and volumes as well as estimates of inter-individual variability. Individual estimates of clearances and volumes will be obtained from post hoc estimates and used to estimate exposure in the central compartment and a peripheral compartment representing the sputum. The change in lung function measured by FEV1 will be summarized descriptively. The proportion of *P. aeruginosa* isolates susceptible to each phage and the phage mixture will be evaluated. The difference between phage and placebo will be calculated, along with a 95% confidence interval.

The Cystic Fibrosis Questionnaire-Revised will be administered at the baseline and final visit for stage 2 participants. This questionnaire contains a series of questions regarding several physical health and ability domains. The Cystic Fibrosis Respiratory Symptom Diary will be collected at all visits for stage 2 participants. Change from baseline through day 30 follow-up visit in Cystic Fibrosis Respiratory Symptom Diary score will be presented by treatment group.

### Methods in analysis to handle protocol non-adherence and any statistical methods to handle missing data {20c}

The primary outcome will be analyzed on the intention-to-treat population. Endpoints may be missing for volunteers who withdraw from the trial. The reasons for withdrawal will be reported and compared by arm. The effect that any missing data might have on results will be assessed via a sensitivity analysis. Baseline characteristics of participants with missing data will be compared to participants without missing data.

### Plans to give access to the full protocol, participant-level data, and statistical code {31c}

The datasets analyzed during the current study and statistical code will be available from the corresponding author, upon reasonable request. The full protocol will be available on clinicaltrials.gov.

## Oversight and monitoring

### Composition of the coordinating center and trial steering committee {5d}

DMID within the NIH serves as the overall study sponsor, responsible for trial conduct and safety oversight. The DMID Clinical Research Operations and Management Support team will conduct site training, monitoring, and close oversight of study visits to assure proper adherence to trial protocol and research standards. Monitoring visits will include periodic review of data submission forms, source data verification, adverse event reporting, and consent documentation.

### Composition of the data monitoring committee, its role, and reporting structure {21a}

Safety oversight will be conducted by a DSMB that is an independent group with the necessary expertise. The DSMB will monitor subject safety and advise DMID. The DSMB members will be separate and independent of research personnel participating in this study and will not have scientific, financial, or other conflicts of interest related to this trial. The DSMB will review data at prespecified intervals during the trial and will conduct ad hoc reviews, as appropriate when a halting rule is met or for immediate concerns regarding observations during the trial.

### Adverse event reporting and harms {22}

All AEs will be graded for severity according to the Common Terminology Criteria for Adverse Events Version 5.0 and assessed for the relationship to the study product. The assessment of the AE’s relationship to the study product will be performed by a licensed clinician. An AE is considered an SAE if, in the view of either the site principal investigator or sponsor, it results in any of the following outcomes: death, a life-threatening AE; inpatient hospitalization, a persistent or significant incapacity or substantial disruption of the ability to conduct normal life functions; or a congenital anomaly/birth defect.

### Frequency and plans for auditing trial conduct {23}

Monitoring for this study will be overseen by the DMID Clinical Research Operations and Management Support monitoring contractor. The Clinical Research Operations and Management Support team will conduct periodic site visits throughout the trial, including a review of data collection forms, source data verification, adverse event reporting, and consent documentation. The monitors will also count and view the study product to verify the number of phage vials.

### Plans for communicating important protocol amendments to relevant parties (e.g., trial participants, ethical committees) {25}

Protocol modifications will be submitted for review by participating institutional review boards (IRBs) for approval prior to implementation. Should any amendments alter the study conduct for participants, participants will be notified of the changes and will be requested to sign updated consent forms.

### Dissemination plans {31a}

Study results will be reported in accordance with Consolidated Standards of Reporting Trials guidelines for randomized controlled trials. Results will be submitted to clinicaltrials.gov within 1 year of study completion. The authors plan to submit study results for publication in a peer-reviewed scientific journal and/or present at relevant conferences. No participant-protected health information will be revealed in any publication or presentation.

## Discussion

Phage usage in human medicine in the West currently remains experimental. In recent years, phage administration as an adjunct therapy to systemic antibiotics for the treatment of highly resistant and/or recalcitrant infections under compassionate use conditions has significantly expanded [6]. Several clinical trials investigating the role of phages as anti-infectives in humans have been completed. These trials have yielded conflicting results, making our comprehension of the role of phage therapy incomplete.

To summarize some notable trials: Wright and colleagues conducted a randomized, controlled trial evaluating the efficacy of phages for the treatment of chronic *P. aeruginosa* otitis media in 24 patients in the UK [17]. Enrollment was limited to patients with *P. aeruginosa* strains with susceptibility to at least one phage in a 6-phage mixture. A single topical application of the phage mixture was applied to the affected areas of patients randomized to the treatment arm. The trial was terminated early due to a 30% greater detectable efficacy signal in the treatment arm, without any treatment-related AEs observed.

Another trial evaluated the efficacy of oral phage therapy to reduce the severity and duration of *Escherichia coli* diarrhea in 120 Bangladeshi children [18]. The trial was terminated after an interim analysis indicated a lack of efficacy with phage therapy compared to oral rehydration. *E. coli* susceptibilities to phage were not evaluated. Moreover, it was unclear if *E. coli* was the causative diarrheal pathogen in participating children and protection of oral phage during gastric transit was not provided. No treatment-related AEs were identified.

The treatment of burn-related infections caused by *P. aeruginosa* using a 12-phage cocktail was investigated in 220 patients in France and Belgium [19]. Patients were randomized to either topical phage therapy or silver sulfadiazine cream for 7 days. Topical phage resulted in longer times to reduction in bacterial colony counts compared to silver sulfadiazine cream. PST was not included as part of the inclusion criteria. Additionally, low dosages of phage (as low as 10–100 PFU) and the lack of stability from the large number of phages in the mixture may have contributed to the negative findings of the trial. Few AEs were noted in the trial and it is unclear if any were related to the assigned treatment; no SAEs were observed.

Finally, a clinical trial was conducted on males prone to urinary tract infections awaiting transurethral resection of the prostate [20]. One hundred thirteen patients were randomized to one of the following three arms: (1) bladder instillations with a phage mixture, (2) bladder installations of a placebo, or (3) standard-of-care antibiotic therapy. The urine bacterial burden was similar in all three groups after 7 days of the assigned treatment. Possible explanations for trial findings include the low dosage of phage at 10^4^–10^5^ PFU and the unexpected effectiveness of the mechanical bladder irrigations.

Although several trials have sought to investigate the efficacy of phage therapy, they have all had notable limitations. Important gaps in knowledge that need further investigation include the efficacy of phage therapy; optimal frequency, dosage, and duration of phage administration; limitations in rapid and accurate laboratory testing platforms—including a reference standard that reliably predicts phage susceptibility; an understanding of the frequency of the emergence of phage resistance; the role of the human immune system in phage efficacy; and the comprehensive safety profile of phage therapy. With continued advancements in medical care, complicated bacterial infections will remain challenging. It is essential to determine if the limitless supply of phages is an important adjunct to traditional anti-infectives. The current trial will provide important insight into the efficacy and safety of phage therapy and provide the foundation for future larger, multi-dose phage studies.

### Trial status

The trial enrolled its first subject on October 3, 2022, and recruitment remains active as of the time of printing. It is estimated that recruitment will be completed by January 31, 2024. The most current protocol version number is 20-0001, version 4.0 dated 7 October 2022.

## Data Availability

Upon completion of the study, final de-identified data may be supplied on request, following submission of a plan for data use, approval of the plan by the NIH, and execution of required institutional agreements.

## Abbreviations

AE: Adverse event
ARLG: Antibacterial Resistance Leadership Group
AUC: Area under the curve
CF: Cystic fibrosis
DMID: Division of Microbiology and Infectious Diseases
DOOR: Desirability of Outcome Ranking
FDA: United States Food and Drug Administration
FEV1: Forced expiratory volume in 1 s
IRB: Institutional review board
QoL: Quality of life
mL: Milliliter
MDRGN: Multidrug-resistant gram-negatives
NIAID: National Institute of Allergy and Infectious Diseases
PFU: Plaque-forming units
SAE: Serious adverse event
WRAIR: Walter Reed Army Institute of Research
